# Intensified preoperative chemoradiation by adding oxaliplatin in locally advanced, primary operable (cT3NxM0) rectal cancer: Impact on long-term outcome. Results of the phase II TAKO 05/ABCSG R‑02 trial

**DOI:** 10.1007/s00066-017-1219-5

**Published:** 2017-11-10

**Authors:** P. Kogler, A. F. DeVries, W. Eisterer, J. Thaler, L. Sölkner, D. Öfner, on behalf of TAKO 05/ABCSG R-02 Trial Investigators

**Affiliations:** 1 Department of Visceral, Transplant and Thoracic Surgery, Medical University of Innsbruck, Anichstr. 35, 6020 Innsbruck, Austria; 2 Department of Radio-Oncology, Feldkirch Hospital, Feldkirch, Austria; 3 Division of Oncology, Department of Internal Medicine, Klagenfurt Hospital, Klagenfurt, Austria; 4 Department of Internal Medicine IV, Wels-Grieskirchen Hospital, Wels, Austria; 5 Austrian Breast and Colorectal Cancer Study Group, Vienna, Austria

**Keywords:** Drug therapy, combination, Lymph nodes, Recurrence, Survival, Surgery, Oxaliplatin, Rektumkarzinom, Rezidiv, Überleben, Chirurgie

## Abstract

**Purpose:**

The major goals of preoperative treatment for locally advanced rectal cancers (LARCs) are improvement of local tumor control, tumor downsizing, and downstaging. Modifications with respect to standardized chemoradiation protocol, e. g., integrating oxaliplatin, are realized with the aim of improving primary tumor response and patient outcome.

**Patients and methods:**

In this phase II multicenter study, patients with LARC of the mid- or lower rectum, cT3cNxcM0 as staged by MRI, were included and treated preoperatively with a combination of capecitabine and oxaliplatin following a standardized protocol during radiation. The focus of this long-term analysis was overall (OS) and disease-free survival (DFS).

**Results:**

A total of 60 patients (19 women, 41 men, median age 60.5 years) were initially enrolled, 1 patient was excluded (violation of study protocol), and 1 was patient lost of follow-up, leading to a total of 58 patients for long-term analysis. The 3‑year OS was 85.5%; 3‑year DFS 71.2%. Over time, 15 patients (25.9%) developed tumor recurrence (1 locoregional, 6.7%; 11 distant, 73.3%; 3 locoregional+distant, 20%). Recurrence-specific therapy was planned in the majority of patients, in 9 of 15 patients (60%) with a radical surgical approach. Of these, 4 patients (44.4%) are again tumor-free at the end of investigation. While tumor downsizing (T level) or pathologically complete response did not influence patient survival, lymph node negativity (LNneg) after preoperative chemoradiation showed significant influence.

**Conclusion:**

LNneg after preoperative treatment for LARC significantly influences patient survival. A radical surgical approach for recurrent LARC (locoregional, distant) should be contemplated when possible as we were able to clearly demonstrate its importance and efficacy.

## Zusammenfassung

**Zielsetzung:**

Durch eine neoadjuvante Radiochemotherapie bei Patienten mit einem lokal fortgeschrittenen Rektumkarzinom (LARC) kann die lokale Tumorkontrolle verbessert werden. Ziel einer Intensivierung durch zusätzliche Gabe von Oxaliplatin ist es, die Reaktion des Primärtumors und das Outcome der Patienten zu verbessern.

**Patienten und Methoden:**

Patienten mit einem LARC im mittleren oder unteren Rektum, welches mittels Magnetresonanztomographie (MRT) als cT3M0 klassifiziert wurde, erhielten im Rahmen einer prospektiven Phase-II-Studie während der präoperativen Radiotherapie eine Kombination aus Capecitabin und Oxaliplatin nach einem standardisierten Protokoll. Fokus der Langzeitstudie waren das Gesamt- (OS) und das tumorfreie Überleben (DFS) der Patienten.

**Ergebnisse:**

Insgesamt wurden 60 Patienten (19 Frauen, 41 Männer, medianes Alter 60,5 Jahre) inkludiert. Ein Patient wurde ausgeschlossen (Protokollverstoß) und ein Patient ging im Follow-up verloren, sodass für die Langzeitanalyse 58 Patienten untersucht wurden. Das 3‑Jahre-OS betrug 85,5 %, das 3‑Jahre-DFS bei 71,2 %. Ein Tumorrezidiv entwickelten 15 Patienten (lokoregionär, distant). Eine rezidivspezifische Therapie wurde für die Mehrzahl der Patienten geplant, bei 9 Patienten (9/15; 60 %) mit einem radikal chirurgischen Therapieansatz. Hiermit konnten bei 4 Patienten (44,4 %) erneut Tumorfreiheit und Langzeitüberleben erreicht werden. Während das Tumor-„downsizing“ oder das Erreichen einer kompletten pathologischen Remission (pCR) keinen Einfluss auf das Patientenüberleben zeigten, konnte ein signifikanter Effekt für Patienten ohne Lymphknotenmetastasen nach intensivierter neoadjuvanter Radiochemotherapie nachgewiesen werden.

**Konklusion:**

Ein negativer Lymphknotenstatus nach intensivierter neoadjuvanter Therapie eines LARC hat einen signifikanten Einfluss auf das Patientenüberleben. Wann immer möglich, ist ein radikaler chirurgischer Ansatz in der Rezidivtherapie nach LARC von entscheidender Bedeutung und effektiv.

Preoperative chemoradiation is known to improve local tumor control and leads to tumor downsizing and downstaging, which counts as a surrogate for survival [1]. Numerous settings with respect to applied chemotherapeutic agents and dose regimens (both for chemotherapy and radiotherapy) exist, all pursuing improved patient outcome with minimal therapy-related adverse events (e. g., toxicity) [2]. Following the German trial by Sauer et al. [3], preoperative 5‑fluorouracil (5-FU) and radiotherapy followed by total mesorectal excision (and postoperative adjuvant 5‑FU) became a standard treatment for locally advanced rectal tumors at least in Europe. As infusional 5‑FU needs to be administered as a continuous infusion during radiation, clinically more convenient, tolerable, and efficient agents were developed in recent years, such as oral bioavailable fluoropyrimidines, e. g., capecitabine (Xeloda®). Capecitabine is an oral prodrug, converted into 5‑FU by intracellular thymidine phosphorylase [4]. Phase I and II trials showed its safety and even its superior efficacy with regard to antitumor activity as compared with infusional 5‑FU [5–7]. Moreover, large phase III studies [8–10] showed its similar efficacy and safety as compared with infusional 5‑FU in the preoperative chemoradiation management of rectal cancer patients.

Oxaliplatin, as a third-generation platinum drug that blocks DNA replication and transcription, is known to exhibit radiosensitizing capability and shows synergy to fluoropyrimidines [11]. For this reason and following studies by DeGramont et al. [12] or Andre et al. [13] that demonstrated the superior efficacy of combining fluoropyrimidine and oxaliplatin in the therapy of metastatic colorectal cancer or in an adjuvant setting, studies focused on the combination of infusional or orally administered 5‑FU with oxaliplatin and radiotherapy in the preoperative setting of locally advanced rectal cancer therapy. The goal of combining the two chemotherapeutic agents was to increase downstaging rates and improve patient survival without adding significant toxicity.

Hence, in 2011 we published the first results of a phase II study focusing on the feasibility, efficacy (down-categorization at the T level) and safety of this preoperative chemoradiation regimen [14]. Patients with MRI-staged cT3NxM0 low rectal cancer were included in this trial. Assessment of lymph node status (N status) was not mandatory, given the lack of accurate determination of N status by imaging as a result of inappropriate sensitivity and specificity [14]. The feasibility of the applied regimen was clearly demonstrated, but the addition of oxaliplatin showed limited therapeutic efficacy while in some patients distinctly increasing toxicity without clearly affecting primary tumor response. Therefore, the goal of this amendment was to analyze the local and distant recurrence rates as well as overall and relapse-free survival after long-term follow-up.

## Patients and methods

The study was designed as a multicenter, phase II trial. Details of the study design, method, and patient inclusion and exclusion criteria were published previously [15]. Eligible patients were 18–80 years of age with histologically confirmed rectal adenocarcinoma up to a maximum of 14 cm from the anal verge. Evidence of cT3 disease with or without nodal involvement by MRI (magnetic resonance imaging) was mandatory for patient inclusion. Written informed consent was obtained from all patients.

### Patient evaluation

All patients gave their medical history and underwent physical examination, biopsy, electrocardiography (ECG), complete laboratory tests, and staging studies. The latter included chest and abdominal pelvis CT (computed tomography) scans, a colonoscopy, and endorectal ultrasound and—mandatory in every patient—a pelvic MRI (exact technique published previously [15]).

### Procedures

#### Radiotherapy

All patients received a total dose of 45 Gy, delivered in three or four high-energy photon beams via a three-dimensional conformation technique, in 25 fractions with a daily fraction of 1.8 Gy given 5 days a week for 5 consecutive weeks.

#### Chemotherapy

Capecitabine was administered at a dose of 825 mg/m^2^ twice daily on radiation days from week 1 to 4. Oxaliplatin, at a dose of 50 mg/m^2^, was applied intravenously as a 2 h infusion on days 1, 8, 15, and 22 prior to radiation therapy.

#### Surgery

Surgery was scheduled 2–4 weeks after completion of chemoradiation. Preferred types of radical resection, according to standardized technique, were low anterior resection (LAR), intersphincteric resection, or abdominoperineal excision (APE); all accompanied with total mesorectal excision (TME).

### Statistical analysis

Survival end points were relapse-free survival which was defined as the interval between surgery and the first evidence of locoregional recurrence, distant metastasis, or death from any cause, cancer-specific survival which was defined as the interval between surgery and cancer-related death, and overall survival which was defined as the interval between surgery and death from any cause.

Analyses were performed according to the intention-to-treat (ITT) principle. Median follow-up was calculated by inverse Kaplan–Meier method. Data are graphically depicted using Kaplan–Meier curves and were tested by log-rank tests. Hazard ratios (HRs) and their corresponding 95% confidence intervals (CIs) were estimated by Cox proportional hazards regression. All analyses were calculated using SAS version 9.3 (SAS Institute, Cary, NC, USA).

## Results

From December 2004 to December 2005, a total of 60 patients (19 women, 41 men) with a median age of 60.5 years (range 34–76 years) were enrolled in the study. One patient was excluded because of study protocol violation (no cT3 tumor), leaving 59 patients for initial analysis. Patient characteristics are summarized in Table 1.

**Table 1:** Patients and tumor characteristics

Patients included	60
Excluded (study violation; no cT3 tumor)	1
Male/female (*n*)	40/19
Median age, years (range)	61 (34–76)
WHO performance status, 0/1 (*n*)	54/5
Distance from the dentale line, median cm (range)	5.42 (0–13)
Tumor stage (by MRI), cT3 (*n*)	59
Tumor differentiation, G1–2/G3/not classified (*n*)	41/9/9
Histologic type, adenocarcinoma/mucinous/others (*n*)	50/5/4
Type of surgery, LAR/IR/APE/not available (*n*)	45/2/11/1
Lymph nodes removed, median (*n*) (range)	15 (5–68)

*MRI* magnetic resonance imaging, *LAR* low anterior resection, *IR* intersphincteric resection, *APE* abdominoperineal resection

### Short-term results

A detailed description of all short-term results was published previously [15]. In summary, total chemotherapy doses were received by 90% (oxaliplatin) and 93% (capecitabine) of all study patients. Six patients (10.1%) underwent a reduction in oxaliplatin therapy dose and 13 patients (22.0%) in capecitabine therapy dose, mostly due to diarrhea occurring during weeks 3–4 of treatment. With respect to radiotherapy, total doses received were 97% of planned doses, with dose reduction being performed in 5 patients (8.5%). Surgery was performed in all patients, with a median of 23 days (range 12–54) between the end of chemoradiation and surgery. The administered multimodal regimen was overall well tolerated with 18 grade 3 adverse events in 13 patients (22.0%) and two grade 4 events (arterial hypertension, *n* = 1; diarrhea, *n* = 1) in 2 patients (3.4%). The most frequently reported grade 3/4 adverse events were diarrhea (7/59 patients; 11.9%), vomiting and nausea (5/59 patients; 8.5%), and arterial hypertension (2/59 patients; 3.4%).

### Long-term results

During this long-term follow-up amendment, with a median follow-up of 79.8 months (range 75.9–83.2) since surgery, 1 patient was lost to follow-up, leaving 58 patients for long-term analysis.

#### Tumor recurrence

**Fig. 1:**
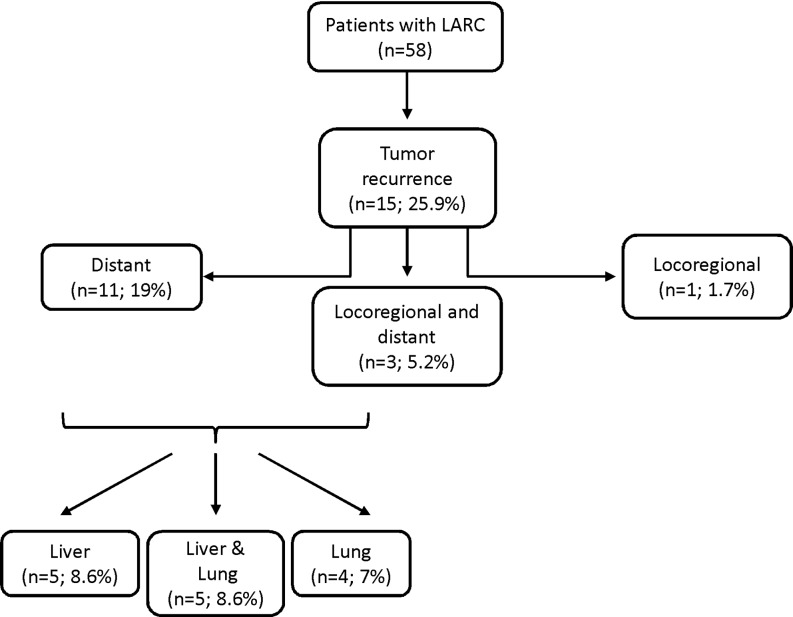
Locally advanced rectal cancer (*LARC*) recurrence: rates and regions (% = *n*/58)

Of the 58 patients, 15 (25.9%) developed tumor recurrence over time (Fig. 1). Of these 15 patients, 1 patient (6.7%) suffered an isolated locoregional recurrence, 11 patients (73.3%) experienced isolated distant metastases, while 3 patients (20%) developed both locoregional and distant recurrences. The most frequent locations for distant recurrences were liver (10/14; 71.4%) and lung (9/14; 64.3%).

#### Tumor recurrence—therapeutic approach

**Fig. 2:**
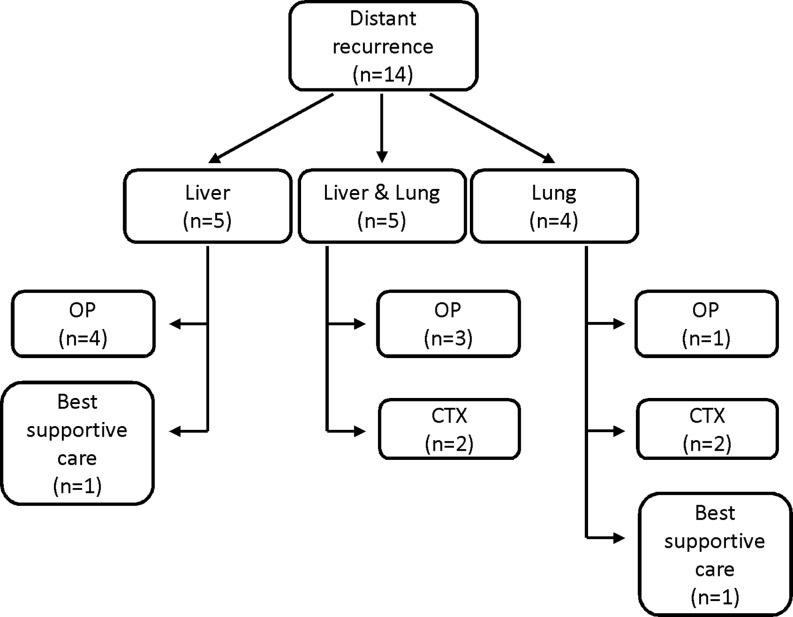
Locally advanced rectal cancers (*LARC*), distant tumor recurrence: therapeutic approach. *OP* surgery, *CTX* chemotherapy

In the majority of patients with diagnosed locoregional and/or distant recurrence a recurrence-specific therapy was conducted (Fig. 2). In detail, in 8 patients with distant recurrence (8/14; 57.1%) and 1 patient with a locoregional recurrence (1/4; 25%), a surgical approach was planned and executed; 6 patients were introduced to systemic therapy (chemotherapy alone) and in 2 patients a combined surgical and chemotherapeutic approach was scheduled.

#### Survival

**Fig. 3:**
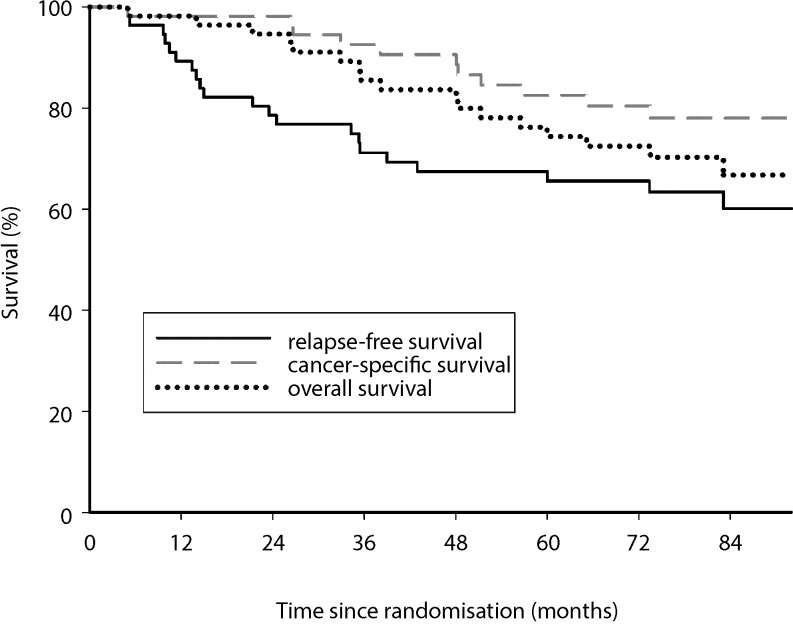
Survival analysis (3 and 5 years): overall survival 85.5 and 74.4%; relapse-free survival 71.2 and 65.6%; cancer-specific survival 92.6 and 82.6%

**Fig. 4:**
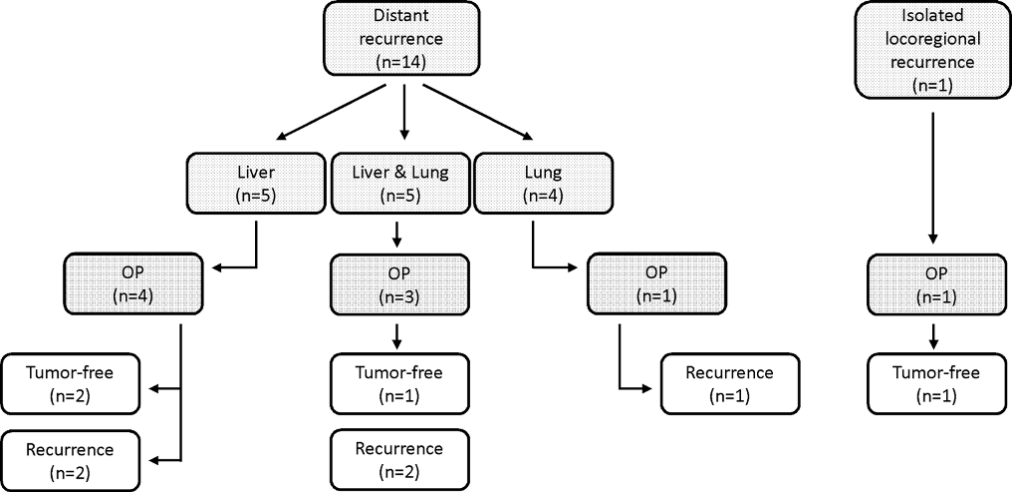
Locally advanced rectal cancer (*LARC*) tumor recurrence: outcome after surgery with curative intent. *OP* surgery

The 3‑ and 5‑year overall survival was 85.5 and 74.4% with a relapse-free survival of 71.2 and 65.5% and a cancer-specific survival of 92.6 and 82.6%, respectively (Fig. 3). Of the 58 patients, 36 patients (62.1%) who underwent initial neoadjuvant chemoradiation with subsequent surgery for LARC were considered tumor-free at the end of the long-term follow-up. Of the 15 patients (15/58; 25.9%) who suffered a locoregional and/or distant recurrence over time, 9 patients (60%) were introduced to a radical surgical approach. Of these, 4 patients (4/9; 44.4%) were again considered tumor-free at the end of follow-up: 1 patient with a verified locoregional recurrence and 3 patients with distant recurrences (2 isolated liver, 1 liver and lung metastases; Fig. 4).

Of the 58 patients, 18 patients died during the investigation period. In 11 patients, the cause of death was associated with initial tumor progress, and 7 patients died due to nontumor-related cause (e. g., cardiac events).

**Fig. 5:**
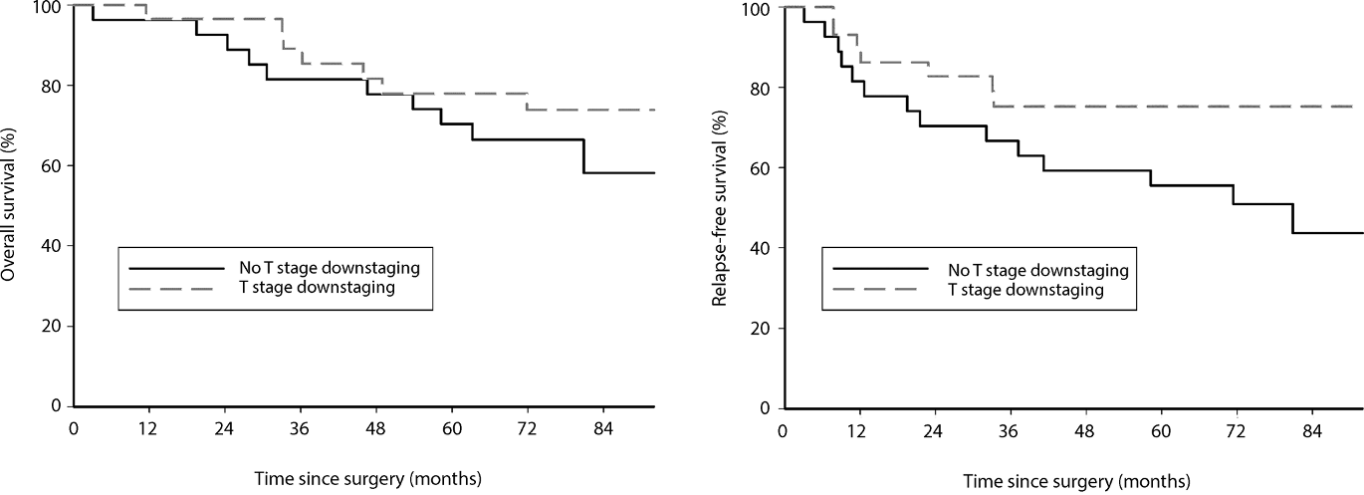
Down-categorization (T-level): no significant influence on overall survival (log rank *p* = 0.3246; DF = 1; Cox *p* = 0.3292), borderline significance on disease-free survival (log rank *p* = 0.0478; DF = 1; Cox *p* = 0.0556)

With reference to the initial study end points, down-categorization at the T level showed no statistically significant influence on overall survival (hazard ratio [HR] 1.61; 95% CI 0.62–4.26; Cox *P* = 0.3292, log rank *P* = 0.3246). With respect to disease-free survival, a borderline significance (HR 2.43; 95% CI 0.98–6.03; Cox *P* = 0.0556, log rank *P* = 0.0478) was demonstrated (Fig. 5). Likewise, complete pathologic response (pCR) after neoadjuvant therapy showed no statistically significant influence on overall (HR 2.20; 95% CI 0.29–16.63; Cox *P* = 0.4437, log rank *P* = 0.4318) or disease-free survival (HR 3.00; 95% CI 0.40–22.40; Cox *P* = 0.2833, log rank *P* = 0.2591) in this patient population.

#### Lymph node status—influence on patient survival

Although assessment of lymph node status at the time of patient enrollment was not mandatory, a review of patient medical charts showed that 40 patients (40/58; 69%) were described as nodal-positive (cN+) at first tumor diagnosis, 9 patients (9/58; 15.5%) as nodal-negative (cN-) and in 9 patients (9/58; 15.5%) nodal status could not be determined. After neoadjuvant therapy, histopathologic workup following surgery showed down-categorization (ypN0 or ypN1 in case of initially cN2, ypN0 in case of initially cN1) in 31 of the 40 initially nodal-positive patients (77.5%). Twenty-eight patients who were initially lymph node-positive (cN+) were now categorized as ypN0 (28/40; 70%). In contrast, 3 of the 9 patients (33.3%) with initially cN− were categorized as ypN+ after histopathologic workup.

**Fig. 6:**
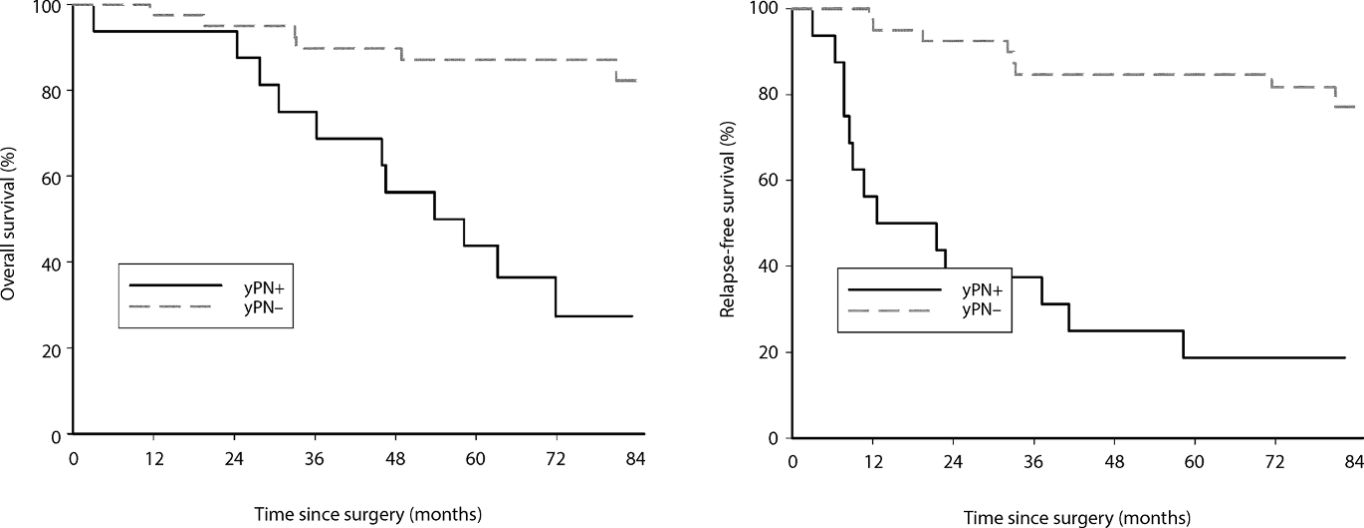
Lymph node negativity after neoadjuvant chemoradiation (*ypN−*) significantly improves overall (log rank *p* < 0.0001; DF = 1; Cox *p* < 0.0001) and disease-free survival (log rank *p* < 0.0001; DF = 1; Cox *p* < 0.0001)

Down-categorization and particularly the achievement of lymph node negativity after neoadjuvant chemoradiation exerts a statistically significant influence on overall 5‑year (HR 6.50; 95% CI 2.36–17.85; Cox *P* = <0.0001, log rank *P* < 0.0001) and disease-free survival (HR 8.13; 95% CI 3.29–20.12; Cox *P* < 0.0001, log rank *P* < 0.0001; Fig. 6).

## Discussion

Therapeutic strategies for LARC have changed distinctly in the past, namely from surgery alone to multimodal approaches, combining surgery with pre- and/or postoperative chemoradiation, thus, realizing the shared aim of local tumor control and high survival rates. Integration of newer generation chemotherapeutics or different application options into the standard treatment plan may further increase tumor control with lower recurrence rates (locally and distant) and, thus, elevate overall and disease-free survival.

Our initial phase II study, investigating a total of 59 patients with LARC, focused on the feasibility, tolerability, and efficacy of oxaliplatin in the preoperative chemoradiation regimen [15]. The intensified protocol was generally well tolerated, with 18 grade 3 adverse events in 13 patients (22.0%) and two grade 4 adverse events in 2 patients (3.4%). Diarrhea was the most common adverse event (12%). These findings correlate with the published literature, reporting grade 3 events ranging from 23–36% and grade 4 events of 3.7% [9, 16, 17]. The primary endpoint of our study was tumor down-categorization with respect to the T level noted as a surrogate for treatment efficacy. Down-categorization (T level) was achieved in 52.5% of our patients with pathologic complete response in 10.1% of our patients. Comparison of these results with the published literature showed that oxaliplatin is not superior to fluorouracil-based chemoradiation protocols [3, 6, 7, 9]. With the exception the German CAO/ARO/AIO-04 trial, which demonstrated a moderate increase in pCR rates with oxaliplatin (17% vs. 13%) an influence of oxaliplatin on primary tumor response (pCR) could not be verified by other randomized trials [9, 18, 19].

Lacking influence on primary tumor response in our study, the goal of the affiliated amendment was to investigate the effects of the intensified treatment regimen on tumor recurrence and patient survival.

With a long median follow-up of 81.81 months, we were able to demonstrate a low incidence of locoregional recurrence for cT3 (MRI-proven) LARC (1.7% vs. 6.9% for isolated or combined recurrence, respectively) in our patient population. Comparison of already published findings shows local recurrence rates with fluorouracil-based preoperative chemoradiation ranging from 5–17% [6, 8, 20–22] and with dose-intensified protocols (+oxaliplatin) from 2.9–11.2% [20, 21, 23]. Despite the influence of the preoperatively administered chemoradiation, excellent surgical technique—good quality of TME, lymphadenectomy, and negative resection margins (R0)—–is mandatory for achieving low locoregional recurrence rates [24]. In our study, no macroscopically incomplete local resection (R2) was performed. Radical surgery with negative resection margins (R0) was achieved in 52 (88.1%) patients, and in 7 (11.9%) patients a microscopically positive resection margin (R1) was detected. This relatively large percentage of R1 resections is clearly due to the primary tumor site being located in the lower rectum and to the locally advanced tumor. While playing an important role in locoregional recurrence, surgery has merely a minor influence on distant recurrence after advanced rectal cancer. Distant recurrence rates of up to 30–40% were seen with fluorouracil-based cancer therapy [3, 8, 21]. Addition of oxaliplatin led to a distant recurrence rate of 19 and 23.3% for isolated or combined (locoregional and distant) recurrence in our patient population. Comparable results are found in the literature, clustering at 22% [20, 21]. Whether the close to 10% risk reduction for distant recurrence is due to the additional application of oxaliplatin in the preoperative regimen cannot be verified from these results, but a contribution to this effect might be suggested.

Management of tumor recurrence is often challenging, requiring an interdisciplinary approach to again achieve a curative setting. In almost 60% of our patients with distant recurrence and 25% of those with locoregional recurrence, a surgical recurrence-specific therapy was planned and carried out; in 2 patients chemotherapy was administered prior to recurrence-specific surgery. It is noteworthy that of these patients undergoing recurrence-specific surgery almost 45% were considered tumor-free at the end of observation. When browsing the literature, a large number of published reports were found on management of liver metastases in colorectal cancer, but only a small number mention the percentage of patients introduced to a curative surgical approach in recurrent disease. Nevertheless, percentages ranged around 20% [25], which is unequivocally distinctly lower than our results. The fact that close to half of our patients with metastatic disease were deemed to be tumor-free at the end of the observation period underscores the importance of a surgical approach and confirms the high standard of care available in Austria [26].

The 5‑year overall survival was 74.4%, 5‑year relapse-free survival 65.5%, and cancer-specific survival 82.6% in our study population. Because of the lack of a control group, it was not possible to analyze the potential influence of oxaliplatin on patient survival in our study population. Addressing this question, controversial results are described in the published trials. While no study was able to identify an influence of oxaliplatin on overall patient survival, the German CAO/ARO/AIO-04 trial [20] reported a significantly higher disease-free survival rate (75.9% vs. 71.2% for the study group [+oxaliplatin] vs. the control group) and the meta-analysis published by Yang et al. [23] a 3-year disease-free survival between 72.7 and 75.9% for the intensified (+oxaliplatin) protocol.

As previously mentioned, the initial end point of our phase II study was tumor down-categorization (T category) and pCR as a surrogate for survival. When addressing these end points and analyzing their effects on survival rates, we were able to show that down-categorization of the T category does not influence overall survival (HR 1.61; 95% CI 0.62–4.26; Cox *P* = 0.3292, log rank *P* = 0.3246), but does influence disease-free survival with borderline significance (HR 2.43; 95% CI 0.98–6.03; Cox *P* = 0.0556, log rank *P* = 0.0478). A larger patient number may be needed to clearly confirm significance. Assessment of the nodal status of the patients included in this study was not mandatory because of the known insufficiency of imaging procedures detecting the lymph nodes involved preoperatively [27, 28]. Nevertheless, after neoadjuvant therapy, down-categorization in the N category was demonstrated in 31 of 40 patients (77.5%), assuming that down-categorization in the T category is accompanied by nodal down-categorization. Similar down-categorization rates were demonstrated by Rödel et al. [17]. The utmost prognostic relevance of lymph node status in patient survival, regardless of the applied chemotherapeutic regimen, was demonstrated previously [29–32]. Likewise, we were able to demonstrate the highly significant influence of lymph node status on patient survival, not only on disease-free but also on overall survival (log rank *P* < 0.0001, Cox *P* < 0.0001).

## Conclusion

Oxaliplatin in the preoperative chemoradiation setting for LARC does not seem to exert a major benefit on primary tumor response or patient survival. If down-categorization (T category, N category) can be achieved, a positive influence on patient survival can be recognized. However, the desired benefit of oxaliplatin could not be fully demonstrated. Nevertheless, the major impact of radical surgery on the treatment plan for and the outcome after distant recurrence following rectal cancer was clearly demonstrated.

## Appendix

In addition to the authors listed on the manuscript, the members of the TAKO 05/ABCSG-R02 were as follows: Medical University of Innsbruck: P. Lukas, R. Jäger, R. Kafka-Ritsch; Hall i.T. Hospital: B. Riedmann; Zams Hospital: P. Sandbichler, E. Wöll; PMU Salzburg: R. Greil, G. Fastner, F. Sedlmayer, B. Mlineritsch, P. Mayer, L. Pleyer, R. Thödtmann, C. Rass, W. Iglseder, G. Kametriser, F. Zehentmayr, M. Kapp, T. Fitzka, A. Dinnewitzer, U. Flatschner, M. Moik, S. Rosenlechner, G. Russ, T. Jäger; Medical University of Graz: K. S. Kapp, R. Schaberl-Moser, H. Samonigg, W. Schippinger; Wels-Grieskirchen Hospital: P. Oppitz, R. Nömeyer, T. Kühr, C. Baldinger, V. Trommet, S. Pillichshammer; St. Veit a.d.G. Hospital: J. Tschmelitsch, H. Weiss, M. Jagoditsch, H.-J. Neumann; Medical University of Vienna: M. Gnant, R. Schmid, F. Herbst, R. Schemed; Wiener Neustadt Hospital: B. Pakitsch, W. Kwasny, P. Wieland, J. Salinger; Leoben Hospital: H. Rabl, G. Suppan, T. Niernberger; Feldkirch Hospital: F. Offner, H. Eiter; BHS Hospital Linz: J. Hammer; Dornbirn Hospital: M. Zitt; Hanusch Hospital Vienna: F. Keil.
